# Exploiting the full power of temporal gene expression profiling through a new statistical test: Application to the analysis of muscular dystrophy data

**DOI:** 10.1186/1471-2105-7-183

**Published:** 2006-04-03

**Authors:** Veronica Vinciotti, Xiaohui Liu, Rolf Turk, Emile J de Meijer, Peter AC 't Hoen

**Affiliations:** 1 Department of Information Systems and Computing, Brunel University, Uxbridge UB8 3PH, UK; 2 Center for Human and Clinical Genetics, Leiden University Medical Center, PO Box 9600, 2300 RC Leiden, Netherlands; 3 Leiden Institute of Advanced Computer Science, Leiden University, PO Box 9512, 2300 RA Leiden, Netherlands; 4 Present affiliation: Howard Hughes Medical Institute, Department of Physiology and Biophysics, Iowa City, Iowa, USA

## Abstract

**Background:**

The identification of biologically interesting genes in a temporal expression profiling dataset is challenging and complicated by high levels of experimental noise. Most statistical methods used in the literature do not fully exploit the temporal ordering in the dataset and are not suited to the case where temporal profiles are measured for a number of different biological conditions. We present a statistical test that makes explicit use of the temporal order in the data by fitting polynomial functions to the temporal profile of each gene and for each biological condition. A Hotelling *T*^2^-statistic is derived to detect the genes for which the parameters of these polynomials are significantly different from each other.

**Results:**

We validate the temporal Hotelling *T*^2^-test on muscular gene expression data from four mouse strains which were profiled at different ages: dystrophin-, beta-sarcoglycan and gamma-sarcoglycan deficient mice, and wild-type mice. The first three are animal models for different muscular dystrophies. Extensive biological validation shows that the method is capable of finding genes with temporal profiles significantly different across the four strains, as well as identifying potential biomarkers for each form of the disease. The added value of the temporal test compared to an identical test which does not make use of temporal ordering is demonstrated via a simulation study, and through confirmation of the expression profiles from selected genes by quantitative PCR experiments. The proposed method maximises the detection of the biologically interesting genes, whilst minimising false detections.

**Conclusion:**

The temporal Hotelling *T*^2^-test is capable of finding relatively small and robust sets of genes that display different temporal profiles between the conditions of interest. The test is simple, it can be used on gene expression data generated from any experimental design and for any number of conditions, and it allows fast interpretation of the temporal behaviour of genes. The R code is available from V.V. The microarray data have been submitted to GEO under series GSE1574 and GSE3523.

## Background

### Introduction

In a typical time course microarray study, a number of microarray experiments are carried out at biologically interesting time points and across different biological conditions. It is a frequent and challenging goal to try to identify which of these genes exhibit an interesting temporal behaviour, for example whether and when a gene becomes up- or down-regulated and, more importantly, whether its behaviour is significantly different across the biological conditions of interest.

Various methods have been proposed in the literature to detect differentially expressed genes from time course microarray experiments. Most of these methods aim at detecting genes whose temporal profile is significantly different from a control condition in which there is no change in expression. Clustering techniques have been long used to analyse time course microarray data with the aim of finding clusters of genes with co-regulated and biologically interesting temporal patterns [1]. Amongst the well known shortcomings of this technique, many clustering methods, like the commonly used hierarchical clustering and k-means, do not make actual use of the temporal order in the data. To address this problem, [2] propose a model-based clustering method for time course data, where each cluster is generated by a vector autoregressive time series model. Other model-based techniques to detect differentially expressed genes from time course microarray data include the use of linear spline functions for single gene profiles by [3] and more specific periodic functions to detect periodically expressed genes by [4] and [5].

Only few studies have looked at the problem of detecting genes with significantly different temporal profiles across a number of biological conditions. [6] use a standard ANOVA analysis. [7] adapt the ANOVA model to account for time ordering by adding three time factors corresponding to the time before, during and after some induction process. Recently, [8] have suggested a method which uses B-splines to model single gene profiles and an appropriate F-test to detect the genes with the fitted profiles significantly different across a number of biological conditions. They validate the method on a biological application to identify genes with different temporal behaviour between treated and untreated cells.

In this paper, we propose a statistical test to detect genes with temporal behaviour significantly different across a number of experimental groups. We account for the temporal order in the data by fitting polynomial models to single gene profiles for each of the groups. We derive a Hotelling *T*^2^-statistic to detect the genes whose fitted profiles are significantly different from each other. We apply the method to the problem of finding genes differentially expressed in four different mouse strains which were profiled at different ages. The biological question and the statistical test are outlined below. The results are presented and extensively discussed in the Results and Discussion section and conclusions are drawn in the Conclusions section The methodology and the data are described in detail in the Methods section.

### Biological question

Muscular dystrophies are a heterogeneous group of inherited neuromuscular disorders characterized by progressive muscle wasting and weakness. A distinct subgroup of muscular dystrophies is caused by mutations in the Dystrophin-associated Glycoprotein Complex (DGC). The DGC is a scaffold protein complex connecting the actin cytoskeleton with the extracellular matrix, thereby contributing to the contractivity of the muscle and the stability of the sarcolemma during contraction. Although genetic mutations in one of the DGC components result in destabilization of the entire complex, the clinical presentation of the diseases associated with these mutations is variable. Dystrophin-deficiency leads to the lethal and highly progressive Duchenne Muscular Dystrophy (DMD), whereas mutations in one of the sarcoglycans result in recessive forms of Limb-Girdle Muscular Dystrophies (LGMD) that usually display a milder phenotype.

A possible explanation for the variable disease phenotypes could be that the proteins involved have other functionalities than just structural roles. It has been suggested that DGC components are also involved in signal transduction routes [9,10] that may be differentially affected by the various deficiencies. To identify such differentially affected processes, we have globally analyzed gene expression profiles in wild-type control mice (abbreviation WT), and mouse models for DMD (dystrophin-deficient mdx mouse [11]; abbreviation MDX), LGMD-2C (gamma-sarcoglycan-deficient mice [12]; abbreviation GSG) and LGMD-2E (beta-sarcoglycan-deficient mice [13]; abbreviation BSG).

This study follows up on a comparison of temporal expression profiles of MDX and WT mice [14] where expression profiles were compared per individual time point and not over the whole time course. Given the fact that there were many genes with expression levels gradually decreasing and increasing in time and a distinct subset of genes for which the expression peaked at the age of 8 weeks, in this paper we propose a statistical test that is more powerful to detect these patterns of expression. In addition, two different mouse models were added, allowing us to compare the disease progression in the different forms of muscular dystrophy. For this reason, the statistical test was developed so as to allow for the comparison of multiple biological conditions.

In order to capture the temporal expression patterns efficiently, a closed temporal loop design was used as a hybridisation design [15]. In these loops, consecutive timepoints were co-hybridised, the last time point being co-hybridised with the first. For each set of 9 individuals from different ages, a separate loop was used, resulting in two loops per strain, and a total of 8 loops and 72 arrays for all four strains.

### Temporal hotelling *T*^2^-test

Given gene expression data on a biological system at a number of time points and under different biological conditions, we wish to detect genes with a temporal profile significantly different between the biological conditions.

Normalised microarray data are summarised as log-ratios of the gene expressions between the two channels on the same array. Depending on the experimental design used to generate the data, these ratios can be either of one condition with respect to a reference sample, if a reference design is used, or of one condition with respect to another, if a loop design is used. In the first step of the modelling process, a simple linear model is considered that converts these observed log-ratios into the parameters of interest that define the gene temporal profile, the true (mean) gene expression with respect to the initial time point.

To account for the temporal order in the data, we model the gene temporal profiles using a polynomial function. As we are interested in detecting genes that peak at a certain time point or genes that are gradually increasing or decreasing over time, we choose to model the temporal profiles using a simple quadratic polynomial. Another reason for using simple temporal models is that they facilitate the biological interpretation of the selected genes, especially when the number of biological conditions is high. Note that, on applications where one is interested in detecting also other temporal behaviour such as periodic temporal profiles, the same methodology described in this paper can also be performed using a polynomial of higher degree.

The linear model on the log-ratios and the polynomial model on the temporal profiles are combined into one single model, from which the maximum likelihood estimates of the parameters, i.e. the coefficients of the polynomials for each biological condition, are obtained. Combining the two models into one, as compared to performing the two-steps sequentially, is an efficient way of obtaining estimates of the parameters of interest, whilst fully accounting for the variation in the data.

Finally, we implement a Hotelling *T*^2^-test to detect the genes for which the coefficients of the polynomial models under the different biological conditions are significantly different, i.e. the genes with a different temporal behaviour across the conditions. For the general case where more than two biological conditions are present, the test can be either done in a pairwise fashion, that is by performing a two-condition test for any pair of conditions, or simultaneously. We show in the results how both options can be successfully utilised to gain insight into genes with significantly different temporal profiles across the conditions. The simultaneous test has the advantage of returning one single list of differentially expressed genes, whereas the pairwise test highlights which pairwise comparison is particularly significant, aiding the discovery of potential biomarkers.

## Results and discussion

### Simulation study

The underlying assumption behind the statistical test proposed in this paper is that by including the knowledge of the temporal ordering in the data the test will aid the detection of genes with interesting and different temporal profiles across the biological conditions.

To test this assumption, we have performed a simulation study where we compare the temporal Hotelling *T*^2^-test with an identical test which does not take into account temporal ordering. Within our framework, this corresponds to performing a Hotelling *T*^2^-test on the estimated gene expression profiles, without fitting a quadratic polynomial to these. This test is very similar to an ANOVA-type of procedure, where the time variable is simply considered as one of the factors, but no knowledge of time ordering is considered.

We have simulated gene expressions for 2 conditions over 8 arrays and 4000 genes. We have generated the data so that 3000 of these genes are not differentially expressed between the two conditions, with their expression drawn from a N
 MathType@MTEF@5@5@+=feaafiart1ev1aaatCvAUfKttLearuWrP9MDH5MBPbIqV92AaeXatLxBI9gBamrtHrhAL1wy0L2yHvtyaeHbnfgDOvwBHrxAJfwnaebbnrfifHhDYfgasaacH8akY=wiFfYdH8Gipec8Eeeu0xXdbba9frFj0=OqFfea0dXdd9vqai=hGuQ8kuc9pgc9s8qqaq=dirpe0xb9q8qiLsFr0=vr0=vr0dc8meaabaqaciaacaGaaeqabaWaaeGaeaaakeaaimaacqWFneVtaaa@383B@(0, *σ*^2^) distribution. The remaining genes are assumed to have different temporal profiles between the two conditions, with the temporal profiles linearly increasing in one condition and decreasing in the other. For all genes, the noise *σ *was varied uniformly between 0.03 and 1.2, whereas the slope of the linear profile for the differentially expressed genes was varied uniformily between 0.005 and 0.2 in absolute value. The range for the noise and slope were chosen to reflect the ones found in our real dataset.

**Figure 1:**
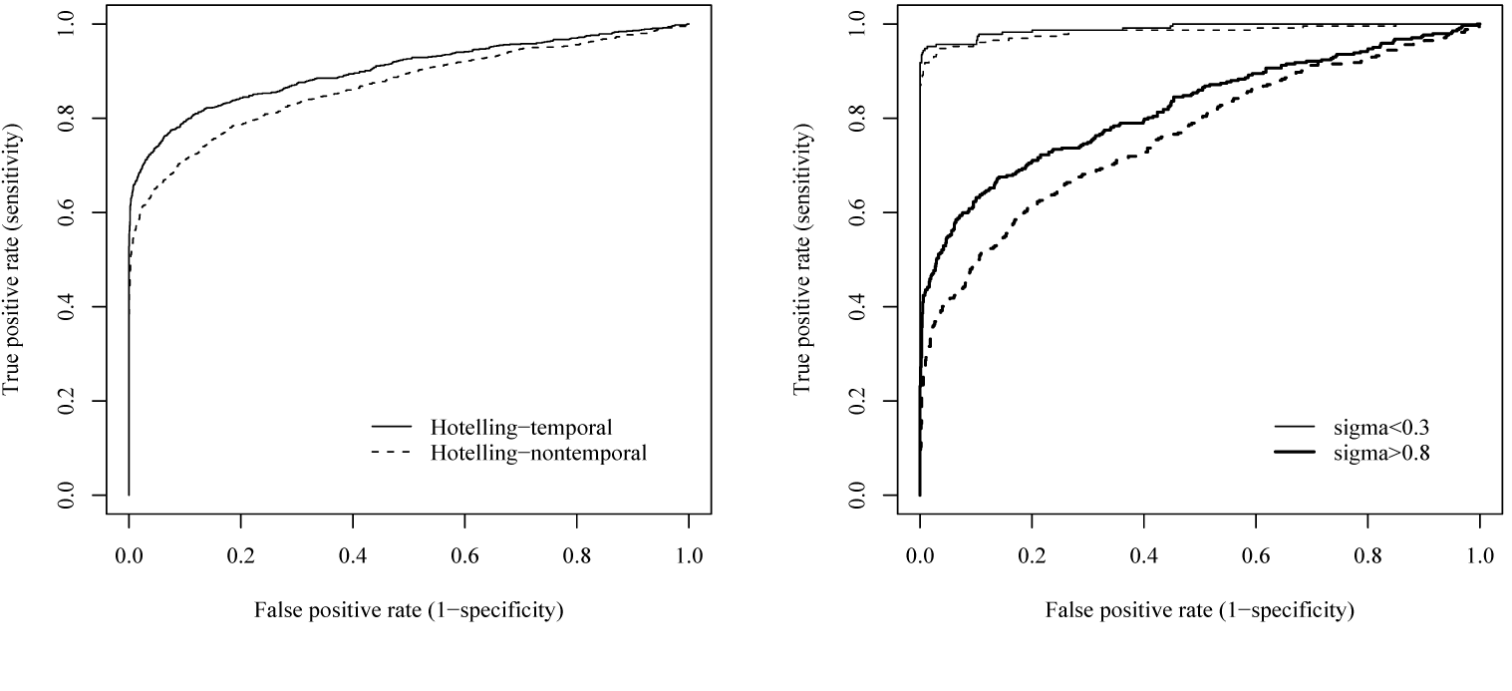
**Simulation study**. ROC curves comparing the temporal Hotelling (solid line) and the non-temporal Hotelling (dotted line) test. The temporal test outperforms the non-temporal one, especially at increasing levels of noise (bold line, right panel).

Figure 1 shows that the temporal test outperforms the non-temporal one, by maximising the percentage of true positives whilst minimizing the percentage of false positives. As expected, by incorporating the knowledge of time using a polynomial and assuming that this model manages to capture the temporal profile, degrees of freedom of the test are saved and the test is more powerful at detecting genes differentially expressed over time. This is pointed out also by [8]. The right panel of Figure 1 shows the performance of the two methods when differentiating the more noisy from the less noisy data. The plot shows how the two tests perform both extremely well when the noise in the data is very low (*σ *< 0.3), but the temporal test shows a clear advantage over the non-temporal test on more noisy data (*σ *> 0.8). That is, the use of a simple polynomial to capture the gene temporal behaviour makes the test more powerful to detect genes with an expression pattern that can be captured by the chosen polynomial.

### Analysis of muscular dystrophy dataset

We have applied the temporal Hotelling *T*^2^-test with second order polynomials on microarray data from wild-type mice and three mouse strains with different forms of muscular dystrophy, to identify genes with differences in temporal expression profiles between the four strains. We compare the results from this test with an identical test that does not take the temporal ordering into account, as described in the previous section.

**Figure 2:**
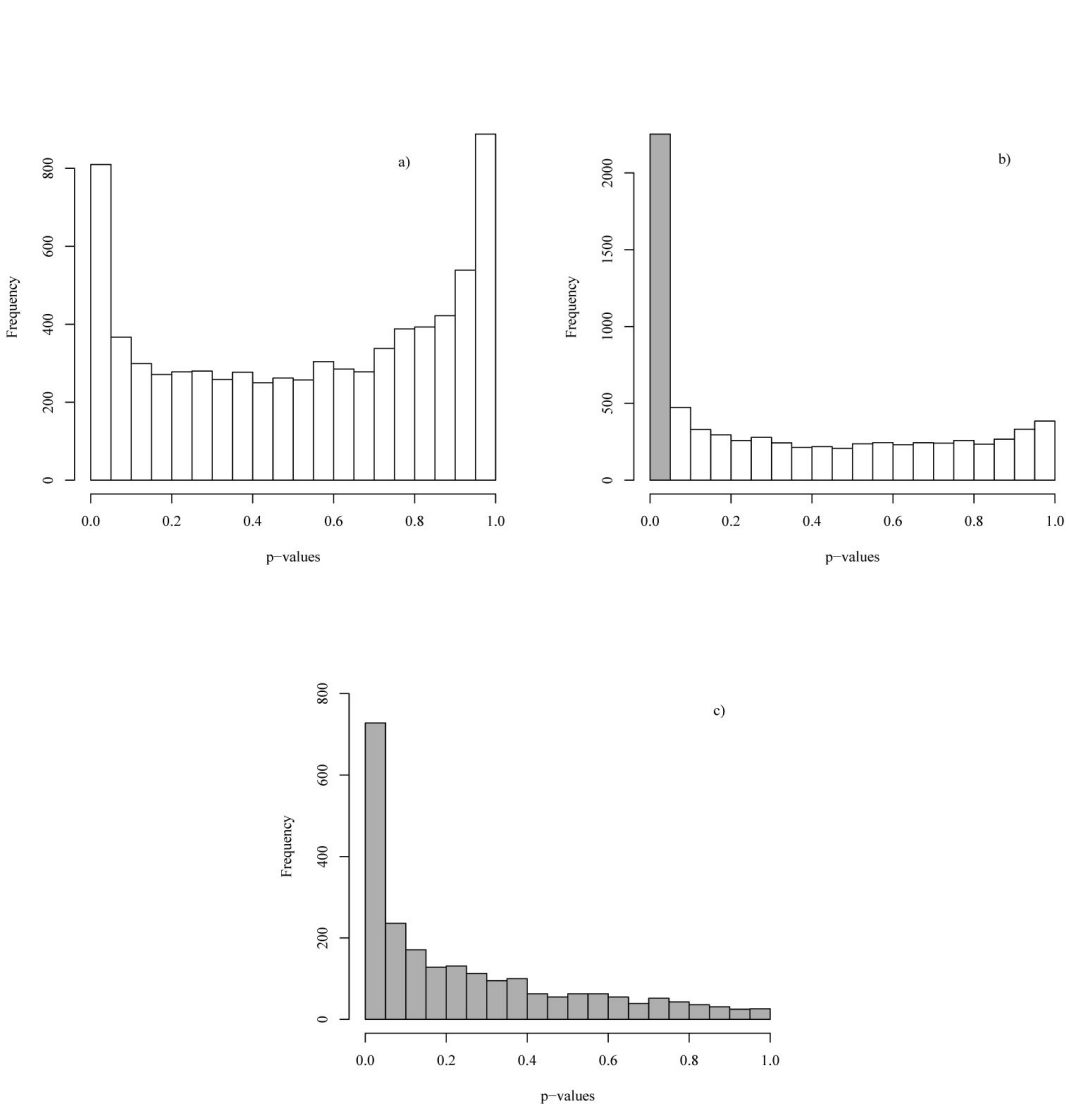
**Distribution of *P*-values**. *P*-value distribution: a) for temporal Hotelling test with polynomial of second degree, b) for non-temporal Hotelling test, c) for the temporal Hotelling test only for genes significant at the 5% confidence level according to the non-temporal test (shaded in grey).

After Benjamini-Hochberg (BH) correction for multiple testing, the temporal test selected 810 genes under 5% confidence. This is lower than the number of genes selected by the non-temporal test (2253). *P-*value distributions for temporal and non-temporal tests are shown in Figures 2a) and 2b), respectively. 728 genes are selected by both tests. In Figure 2c), we show that the remaining genes selected by the non-temporal test display a wide distribution of *P*-values in the temporal test. We hypothesise that these genes show extensive biological variation in expression patterns over time, as was hinted by the simulation study. To demonstrate the validity of this hypothesis, in the next paragraphs, we report on genes which either were found significant by both statistical tests or which had extremely different outcomes in the two tests. We excluded the possibility that the observed differences in temporal profiles between the individual strains were due to technical artefacts arising from the microarray technology. We did so by determining the temporal expression profiles of this selection of genes by the independent quantitative reverse-transcription PCR (qPCR) technique.

**Figure 3:**
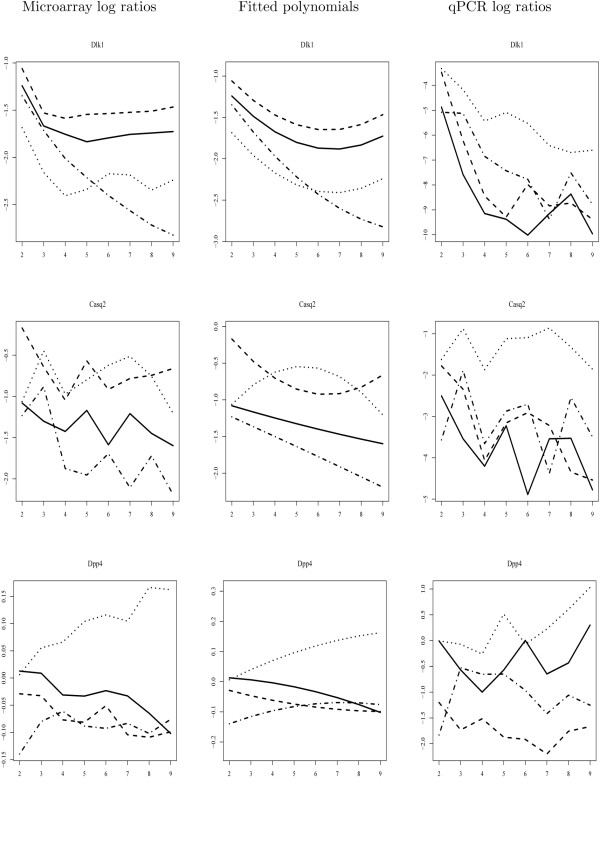
**Validation of the temporal Hotelling *T*^2 ^-test with quantitative PCR experiments (1)**.The expression patterns of *Dlk1 *and *Casq2 *(significant in both temporal and non-temporal tests), and *Dpp4 *(significant in temporal but not in non-temporal test), as determined by microarray and qPCR assays, are plotted. The left panel shows the log-ratios with respect to time point 1 estimated from the microarray data; the middle panel shows the fitted second order polynomials; the right panel shows the log-ratios determined by quantitative PCR, as an independent confirmation method for the microarray data. X-axis represents time points. Solid lines represent WT mice; dashed lines MDX mice; dotted lines BSG mice; dashed line interrupted with dots GSG mice.

Delta-like 1 homolog (Drosophila) (*Dlk1*) was amongst the most significant genes according to both tests (*P*-value < 1E-12). There is a strong decrease in expression of this gene from the age of one week, with the rate at which the expression decreases in the different strains being significantly different (Figure 3, first row, left panel). This is also apparent in the plot of the fitted polynomials (middle panel). The trend was confirmed by the qPCR assay (right panel). However, in the qPCR assay, an even sharper drop in expression from the first to the second time point is observed, also explaining the differences in heights of the curves in the microarray and qPCR experiments. Interestingly, also the *Gtl2 *gene (the murine homologue of the human *MEG3 *gene), which is imprinted reciprocally to *Dlk1*, demonstrates prolonged expression in mouse models for muscular dystrophies (especially in MDX mice, as shown also by the pairwise tests of this strain against the others). In sheep, a mutation in the region between the imprinted *DLK1 *and *MEG3 *genes gives rise to prolonged postnatal expression of *DLK1*, and the callipyge muscular hypertrophy phenotype [16,17]. An elevation of hypertrophic fibres is therefore a likely phenotype of the mouse models analysed, and has been established as a hallmark of DMD [18].

Like *Dlk1*, Calsequestrin 2 (*Casq2*) is selected with high confidence according to both tests. The qPCR assay confirms its differential expression (Figure 3, second row). However, as for the case of *Dlk1*, the similarity in the shape of the curves between the microarray and qPCR experiments is not comfirmed at the level of the ratio values themselves. This is probably due to differences in the assay set-up and normalisation procedures.

Dipeptidylpeptidase 4 (*Dpp4*) was highly ranked in the temporal test (232) but not in the non-temporal test (2710). This is likely to be caused by the fact that the changes in gene expression are minor but consistent in time (Figure 3, third row). Through evaluation of the pairwise comparisons between each strain utilizing the same temporal test, it appeared that the *P-*values for every comparison with the BSG model were 0.03, whereas *P*-values in other pairwise comparisons were greater than 0.3. This indicates that the behaviour of this gene in BSG is different from the other animal models, which would make it a biomarker for BSG-deficiency. The up-regulation of *Dpp4 *in BSG but not in the other mouse models was confirmed by qPCR (Figure 3, third row, right panel), and demonstrates the power of the test in the detection of small but consistent temporal changes. Dpp4 (CD26) plays a role in antigen-specific T-cell activation [19], a process which might contribute to the severe phenotype of BSG mice.

**Figure 4:**
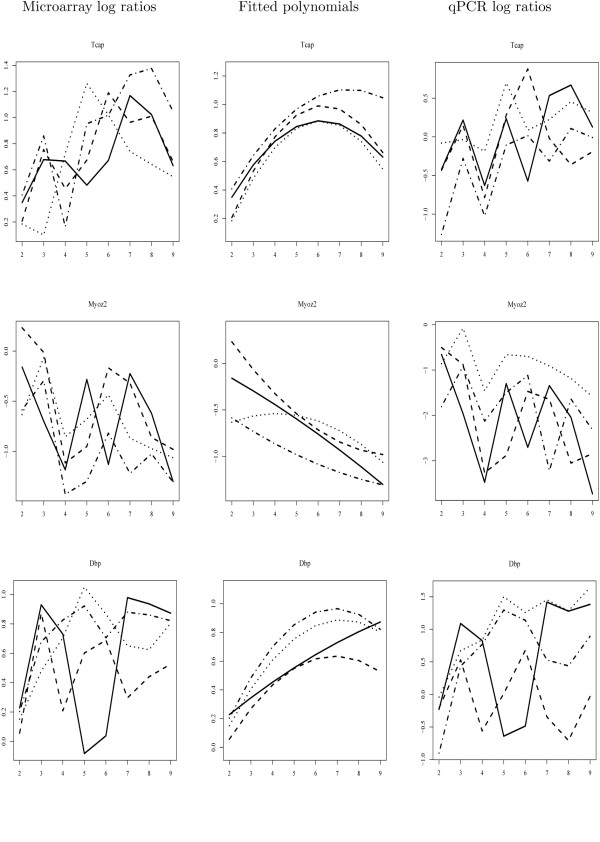
**Validation of the temporal Hotelling *T*^2^-test with quantitative PCR experiments (2)**. The expression patterns of *Tcap *and *Myoz2 *(significant in non-temporal but not in temporal test), and *Dbp *(significant in non-temporal test and temporal test with higher order polynomials), as determined by microarray and qPCR assays, are plotted. The left panel shows the log-ratios with respect to time point 1 estimated from the microarray data; the middle panel shows the fitted second order polynomials; the right panel shows the log-ratios determined by quantitative PCR, as an independent confirmation method for the microarray data. X-axis represents time points. Solid lines represent WT mice; dashed lines MDX mice; dotted lines BSG mice; dashed line interrupted with dots GSG mice.

Titin-cap (telethonin; *Tcap*) is not selected by the temporal test despite very low *P*-values (2.5E-13) in the non-temporal test. *Tcap *is a potentially important gene since *Tcap *mutations result in Limb-Girdle Muscular Dystrophy 2G [20]. We observed a gradual increase over time in all the animal models, including wild-type animals. However, a significant difference in temporal behaviour between mouse models was not found, and both microarray and qPCR experiments demonstrated high overall variability. This is smoothed-out in the fitted polynomials, which are highly similar for the four mouse models (Figure 4, first row, middle panel). We therefore think that differences in observed expression levels are caused by inter-individual variation rather than by the dystrophic process. Similarly, Myozenin2 (*Myoz2*)(*P*-value 0.71 and < 1E-12 for temporal and non-temporal test, respectively) was confirmed to demonstrate high variability across time points in the qPCR experiment (Figure 4, second row). The temporal test seems to be better able to filter out genes with high inter-individual variation when low numbers of replicates are used, as was the case for our study (two animals per time point per strain).

D site albumin promoter binding protein (*Dbp*) and its downstream target Periodically expressed 1 (*Per1*) were not identified as differentially expressed using the model with the second-order polynomial, but were significant when higher order polynomials were applied, and were also significant according to the non-temporal test. The wave-like expression patterns observed in the time series of MDX and WT mice were also found in the qPCR experiment (Figure 4, third row). Mentioned genes have been found to be regulated by the circadian clock [21]. They are not related to the muscular dystrophy but their differential expression is likely to be caused by the time of the day at which the animals were sacrificed. Since littermates were used for most timepoints, the time point at which the animals were sacrificed was consistent for a certain strain and age, but variable for animals of different strains and ages. This example shows that our temporal test with higher order polynomials can be successfully used to find genes with more complex temporal expression patterns, if this is of interest to the experimenter.

The coefficients of the fitted polynomials can be used to identify biomarkers for muscular dystrophy in general and for the individual strains, by looking at genes with some interesting specified temporal pattern. As an example, we made a selection of significant genes with the quadratic coefficient less than -0.02 in MDX, BSG and GSG but not in WT. When fitting a parabolic function, the quadratic coefficient reflects the extreme of the curve. When the quadratic coefficient is negative the extreme is a maximum, corresponding with (temporary) upregulation. We ended up with 11 genes that were all related to inflammation *(Ccl6, Ccl8, Ccl9, Clecsf12, Ii, Laptm5, Lgals3, Lzp-s, Mpeg1, Ptpns1, S100a4*). Without exceptions, these genes were found to be up-regulated in an independent comparative study of mouse models for muscular dystrophy (profiled only at the age of 8 weeks, [22]) with BH-corrected *P-*values ranging from 4E-4 to 4E-14. This observation confirms the important contribution of inflammation to the dystrophic phenotype in dystrophin-and sarcoglycan-deficient mice.

As an alternative to the use of the polynomial coefficients to filter out interesting genes amongst the significant ones, one can detect potential biomarker genes for a certain strain by looking at the genes that have significant *P*-values in the pairwise comparisons of this particular strain with all other strains. Table 1 shows a list of potential biomarker genes identified at the 5% confidence level. *Gtl2 *and *Dpp4 *are found as potential biomarkers for MDX and BSG, as already observed above. Fibulin 5 (*FblnS*) is found as a biomarker for MDX. In contrast to WT, BSG and GSG, where the expression of *Fbln5 *gradually decreases over time, MDX mice show an initial up-regulation, followed by a sharp reduction in expression at the age of 2.5 weeks. This results in lower levels of expression in MDX mice than in the other strains from the age of 6 weeks. Since Fbln5 seems to be an inhibitor of vascular smooth muscle cell proliferation and migration [23], the observed profile in MDX may hint at increased vascular remodelling activity in these mice, contributing to the higher regenerative activity, compared to other models. *Atp5j*, which encodes for a component of the mitochondrial ATP synthase complex, demonstrates a sharp drop in expression in BSG mice at 6 and 8 weeks of age but not in other animals. This may be indicative of a temporal metabolic crisis in BSG. Matrilin2 (*Matn2*) is much higher expressed in GSG mice aged 1 week than in other mice, and remains more highly expressed than in WT mice over the whole time course. As also evident from the many extracellular matrix proteins, such as collagens, decorin, and biglycan, found differentially expressed in previous gene expression profiling studies in muscular dystrophies [24-27], the differential expression of *Matn2 *reflects the extensive ongoing extracellular matrix remodeling in the diseases.

## Conclusion

In most of the analyses of temporal gene expression profiling experiments, the full power of the temporal relationship in the data is not exploited, because not many models include the information of temporal order. This paper describes a simple statistical method that incorporates this information, thereby enabling the identification of genes that show consistent differences in temporal behaviour between a number of biological conditions. The findings from the statistical analysis were extensively validated using a simulation study, quantitative PCR, and a review of the biological function of the identified genes.

The performance of the test was compared to the performance of an identical test that does not take temporal ordering into account. The latter test identified many more genes, but mainly genes that demonstrated more "noisy" temporal profiles, which may not be of much interest since they are mainly caused by large variability between animals and time points. In our study, the smoothing effect of the quadratic polynomial fit is probably essential to filter out these "noisy" gene profiles and select genes with consistent differences between strain. Therefore, the described test makes it possible to capture simple trends in the expression of genes using a limited number of biological replicates. More difficult trends can be captured using higher order polynomials, but require more biological replicates and higher sampling rates to avoid overfitting of the data. The same is probably true for the fitting of non-polynomial functions like sigmoids or exponentials, which would have the advantage of better biological interpretability of the coefficients. In addition, non-polynomial functions would require a different type of statistics since the coefficients are not normally distributed and therefore violate the assumptions of the currently presented test.

The temporal Hotelling *T*^2^-test presented in this paper can be used in any experimental design and for any number of conditions. In this study, however, we have applied it to data coming from a loop design in which subsequent time points were co-hybridized, thereby putting even more emphasis on the temporal relationships in the data. Therefore, the described experimental design and data analysis method constitute an important step in the optimisation of temporal expression profiling efforts, both in terms of efficiency (requiring as few arrays as possible) and statistical performance.

## Methods

### Linear model for contrasts estimation

Let ytjtkc
 MathType@MTEF@5@5@+=feaafiart1ev1aaatCvAUfKttLearuWrP9MDH5MBPbIqV92AaeXatLxBI9gBaebbnrfifHhDYfgasaacH8akY=wiFfYdH8Gipec8Eeeu0xXdbba9frFj0=OqFfea0dXdd9vqai=hGuQ8kuc9pgc9s8qqaq=dirpe0xb9q8qiLsFr0=vr0=vr0dc8meaabaqaciaacaGaaeqabaqabeGadaaakeaacqWG5bqEdaqhaaWcbaGaemiDaq3aaSbaaWqaaiabdQgaQbqabaWccqWG0baDdaWgaaadbaGaem4AaSgabeaaaSqaaiabdogaJbaaaaa@35B1@ denote the log-ratio between the time *t*_*j *_and time *t*_*k *_hybridised on the same array under the biological condition *c *and for a particular gene. For simplicity, we avoid referring to the specific gene in the notation. Let μ1tc
 MathType@MTEF@5@5@+=feaafiart1ev1aaatCvAUfKttLearuWrP9MDH5MBPbIqV92AaeXatLxBI9gBaebbnrfifHhDYfgasaacH8akY=wiFfYdH8Gipec8Eeeu0xXdbba9frFj0=OqFfea0dXdd9vqai=hGuQ8kuc9pgc9s8qqaq=dirpe0xb9q8qiLsFr0=vr0=vr0dc8meaabaqaciaacaGaaeqabaqabeGadaaakeaaiiGacqWF8oqBdaqhaaWcbaGaeGymaeJaemiDaqhabaGaem4yamgaaaaa@3246@ denote the true mean gene expression at time *t *with respect to the initial time point and under condition *c*. The vector *μ*^*c *^= (μ12c,…,μ1Tc)
 MathType@MTEF@5@5@+=feaafiart1ev1aaatCvAUfKttLearuWrP9MDH5MBPbIqV92AaeXatLxBI9gBaebbnrfifHhDYfgasaacH8akY=wiFfYdH8Gipec8Eeeu0xXdbba9frFj0=OqFfea0dXdd9vqai=hGuQ8kuc9pgc9s8qqaq=dirpe0xb9q8qiLsFr0=vr0=vr0dc8meaabaqaciaacaGaaeqabaqabeGadaaakeaadaqadaqaaGGaciab=X7aTnaaDaaaleaacqaIXaqmcqaIYaGmaeaacqWGJbWyaaGccqGGSaalcqWIMaYscqGGSaalcqWF8oqBdaqhaaWcbaGaeGymaeJaemivaqfabaGaem4yamgaaaGccaGLOaGaayzkaaaaaa@3B94@ for all time points *t *is the true temporal profile for a specific gene under the biological condition *c*. This is what we wish to estimate from the available data.

For each gene and under a wide range of circumstances [28], the vector *y*^*c *^of all the log-ratios ytjtkc
 MathType@MTEF@5@5@+=feaafiart1ev1aaatCvAUfKttLearuWrP9MDH5MBPbIqV92AaeXatLxBI9gBaebbnrfifHhDYfgasaacH8akY=wiFfYdH8Gipec8Eeeu0xXdbba9frFj0=OqFfea0dXdd9vqai=hGuQ8kuc9pgc9s8qqaq=dirpe0xb9q8qiLsFr0=vr0=vr0dc8meaabaqaciaacaGaaeqabaqabeGadaaakeaacqWG5bqEdaqhaaWcbaGaemiDaq3aaSbaaWqaaiabdQgaQbqabaWccqWG0baDdaWgaaadbaGaem4AaSgabeaaaSqaaiabdogaJbaaaaa@35B1@ under condition *c *can be represented in matrix form as

*y*^*c *^= *X μ*^*c *^+ *ε*^*c*^,     (1)

where *X *is the *design matrix *defining the relationship between the values observed in the experiment and the parameters of interest, *μ*^*c *^and *ε*^*c *^is a vector of independent, normally distributed, zero-mean errors with variance σc2
 MathType@MTEF@5@5@+=feaafiart1ev1aaatCvAUfKttLearuWrP9MDH5MBPbIqV92AaeXatLxBI9gBaebbnrfifHhDYfgasaacH8akY=wiFfYdH8Gipec8Eeeu0xXdbba9frFj0=OqFfea0dXdd9vqai=hGuQ8kuc9pgc9s8qqaq=dirpe0xb9q8qiLsFr0=vr0=vr0dc8meaabaqaciaacaGaaeqabaqabeGadaaakeaaiiGacqWFdpWCdaqhaaWcbaGaem4yamgabaGaeGOmaidaaaaa@30E4@. This general model is appropriate for any design used in the experiments, whether it is a reference or a loop design, as the information on the design is encoded by the matrix *X *[29]. For the case of a reference design, the log-ratios *y^c ^*will be with respect to a reference sample and similarly the parameters of interest *μ^c^*.

### Polynomial fitting of gene temporal profile

We aim to capture the general temporal behaviour of a gene under a specific condition using a polynomial function of degree *p*. This corresponds to assuming that

μ1tc=α0c+α1ct+…+αpctp.     (2)
 MathType@MTEF@5@5@+=feaafiart1ev1aaatCvAUfKttLearuWrP9MDH5MBPbIqV92AaeXatLxBI9gBaebbnrfifHhDYfgasaacH8akY=wiFfYdH8Gipec8Eeeu0xXdbba9frFj0=OqFfea0dXdd9vqai=hGuQ8kuc9pgc9s8qqaq=dirpe0xb9q8qiLsFr0=vr0=vr0dc8meaabaqaciaacaGaaeqabaqabeGadaaakeaaiiGacqWF8oqBdaqhaaWcbaGaeGymaeJaemiDaqhabaGaem4yamgaaOGaeyypa0Jae8xSde2aa0baaSqaaiabicdaWaqaaiabdogaJbaakiabgUcaRiab=f7aHnaaDaaaleaacqaIXaqmaeaacqWGJbWyaaGccqWG0baDcqGHRaWkcqWIMaYscqGHRaWkcqWFXoqydaqhaaWcbaGaemiCaahabaGaem4yamgaaOGaemiDaq3aaWbaaSqabeaacqWGWbaCaaGccqGGUaGlcaWLjaGaaCzcamaabmaabaGaeGOmaidacaGLOaGaayzkaaaaaa@4CEA@

In matrix form, for all time points *t*, this can be written as

*μ*^*c *^= *A α*^*c*^,

where αc=(α0c,…,αpc)
 MathType@MTEF@5@5@+=feaafiart1ev1aaatCvAUfKttLearuWrP9MDH5MBPbIqV92AaeXatLxBI9gBaebbnrfifHhDYfgasaacH8akY=wiFfYdH8Gipec8Eeeu0xXdbba9frFj0=OqFfea0dXdd9vqai=hGuQ8kuc9pgc9s8qqaq=dirpe0xb9q8qiLsFr0=vr0=vr0dc8meaabaqaciaacaGaaeqabaqabeGadaaakeaaiiGacqWFXoqydaahaaWcbeqaaGqaciab+ngaJbaakiabg2da9maabmaabaGae8xSde2aa0baaSqaaiabicdaWaqaaiabdogaJbaakiabcYcaSiablAciljabcYcaSiab=f7aHnaaDaaaleaacqWGWbaCaeaacqWGJbWyaaaakiaawIcacaGLPaaaaaa@3DE6@ and *A *is the matrix of time entries. This combined to equation (1) leads to the final formula

*y*^*c *^= *X Aα*^*c *^+ *ε*^*c*^.     (3)

From the theory of linear models, the maximum likelihood estimate of *α*^*c *^from the model (3) is given by

α^c=(X˜tX˜)−1X˜tyc,
 MathType@MTEF@5@5@+=feaafiart1ev1aaatCvAUfKttLearuWrP9MDH5MBPbIqV92AaeXatLxBI9gBaebbnrfifHhDYfgasaacH8akY=wiFfYdH8Gipec8Eeeu0xXdbba9frFj0=OqFfea0dXdd9vqai=hGuQ8kuc9pgc9s8qqaq=dirpe0xb9q8qiLsFr0=vr0=vr0dc8meaabaqaciaacaGaaeqabaqabeGadaaakeaaiiGacuWFXoqygaqcamaaCaaaleqabaGaem4yamgaaOGaeyypa0ZaaeWaaeaacuWGybawgaacamaaCaaaleqabaGaemiDaqhaaOGafmiwaGLbaGaaaiaawIcacaGLPaaadaahaaWcbeqaaiabgkHiTiabigdaXaaakiqbdIfayzaaiaWaaWbaaSqabeaacqWG0baDaaGccqWG5bqEdaahaaWcbeqaaiabdogaJbaakiabcYcaSaaa@3F94@

where X˜
 MathType@MTEF@5@5@+=feaafiart1ev1aaatCvAUfKttLearuWrP9MDH5MBPbIqV92AaeXatLxBI9gBaebbnrfifHhDYfgasaacH8akY=wiFfYdH8Gipec8Eeeu0xXdbba9frFj0=OqFfea0dXdd9vqai=hGuQ8kuc9pgc9s8qqaq=dirpe0xb9q8qiLsFr0=vr0=vr0dc8meaabaqaciaacaGaaeqabaqabeGadaaakeaacuWGybawgaacaaaa@2DF4@ = *X A*.

### Temporal hotelling *T*^2^-test for two conditions

The aim of the analysis is to detect genes for which the temporal profiles across the different biological conditions are significantly different to each other. To do so, we look for significant differences between the parameters of the estimated polynomials, the α^c
 MathType@MTEF@5@5@+=feaafiart1ev1aaatCvAUfKttLearuWrP9MDH5MBPbIqV92AaeXatLxBI9gBaebbnrfifHhDYfgasaacH8akY=wiFfYdH8Gipec8Eeeu0xXdbba9frFj0=OqFfea0dXdd9vqai=hGuQ8kuc9pgc9s8qqaq=dirpe0xb9q8qiLsFr0=vr0=vr0dc8meaabaqaciaacaGaaeqabaqabeGadaaakeaaiiGacuWFXoqygaqcamaaCaaaleqabaGaem4yamgaaaaa@2FDE@, where *c *= 1,...,*K *and *K *is the number of experimental groups.

We first consider the case where *K *= 2. This is the most occurring case in the literature, where one aims at detecting differences between a control group and a treated group over time. We use a Hotelling *T*^2^-test to detect significant differences between the parameters α^1
 MathType@MTEF@5@5@+=feaafiart1ev1aaatCvAUfKttLearuWrP9MDH5MBPbIqV92AaeXatLxBI9gBaebbnrfifHhDYfgasaacH8akY=wiFfYdH8Gipec8Eeeu0xXdbba9frFj0=OqFfea0dXdd9vqai=hGuQ8kuc9pgc9s8qqaq=dirpe0xb9q8qiLsFr0=vr0=vr0dc8meaabaqaciaacaGaaeqabaqabeGadaaakeaaiiGacuWFXoqygaqcamaaCaaaleqabaGaeGymaedaaaaa@2F7F@ and α^2
 MathType@MTEF@5@5@+=feaafiart1ev1aaatCvAUfKttLearuWrP9MDH5MBPbIqV92AaeXatLxBI9gBaebbnrfifHhDYfgasaacH8akY=wiFfYdH8Gipec8Eeeu0xXdbba9frFj0=OqFfea0dXdd9vqai=hGuQ8kuc9pgc9s8qqaq=dirpe0xb9q8qiLsFr0=vr0=vr0dc8meaabaqaciaacaGaaeqabaqabeGadaaakeaaiiGacuWFXoqygaqcamaaCaaaleqabaGaeGOmaidaaaaa@2F81@. This is equivalent to testing whether the parameter β^=α^1−α^2
 MathType@MTEF@5@5@+=feaafiart1ev1aaatCvAUfKttLearuWrP9MDH5MBPbIqV92AaeXatLxBI9gBaebbnrfifHhDYfgasaacH8akY=wiFfYdH8Gipec8Eeeu0xXdbba9frFj0=OqFfea0dXdd9vqai=hGuQ8kuc9pgc9s8qqaq=dirpe0xb9q8qiLsFr0=vr0=vr0dc8meaabaqaciaacaGaaeqabaqabeGadaaakeaaiiGacuWFYoGygaqcaiabg2da9iqb=f7aHzaajaWaaWbaaSqabeaacqaIXaqmaaGccqGHsislcuWFXoqygaqcamaaCaaaleqabaGaeGOmaidaaaaa@35F1@ is significantly different from zero. The distribution of this estimate can be derived under our model assumptions. From Equation (3), it follows that α^c~N(αc,(X˜tX˜)−1σc2)
 MathType@MTEF@5@5@+=feaafiart1ev1aaatCvAUfKttLearuWrP9MDH5MBPbIqV92AaeXatLxBI9gBaebbnrfifHhDYfgasaacH8akY=wiFfYdH8Gipec8Eeeu0xXdbba9frFj0=OqFfea0dXdd9vqai=hGuQ8kuc9pgc9s8qqaq=dirpe0xb9q8qiLsFr0=vr0=vr0dc8meaabaqaciaacaGaaeqabaqabeGadaaakeaaiiGacuWFXoqygaqcamaaCaaaleqabaGaem4yamgaaOGaeiOFa4NaemOta4KaeiikaGIae8xSde2aaWbaaSqabeaacqWGJbWyaaGccqGGSaalcqGGOaakcuWGybawgaacamaaCaaaleqabaGaemiDaqhaaOGafmiwaGLbaGaacqGGPaqkdaahaaWcbeqaaiabgkHiTiabigdaXaaakiab=n8aZnaaDaaaleaacqWGJbWyaeaacqaIYaGmaaGccqGGPaqkaaa@4477@. Assuming independence between the parameters *α*^*c *^for different conditions, it follows that the estimate β^
 MathType@MTEF@5@5@+=feaafiart1ev1aaatCvAUfKttLearuWrP9MDH5MBPbIqV92AaeXatLxBI9gBaebbnrfifHhDYfgasaacH8akY=wiFfYdH8Gipec8Eeeu0xXdbba9frFj0=OqFfea0dXdd9vqai=hGuQ8kuc9pgc9s8qqaq=dirpe0xb9q8qiLsFr0=vr0=vr0dc8meaabaqaciaacaGaaeqabaqabeGadaaakeaaiiGacuWFYoGygaqcaaaa@2E64@ is distributed according to β^
 MathType@MTEF@5@5@+=feaafiart1ev1aaatCvAUfKttLearuWrP9MDH5MBPbIqV92AaeXatLxBI9gBaebbnrfifHhDYfgasaacH8akY=wiFfYdH8Gipec8Eeeu0xXdbba9frFj0=OqFfea0dXdd9vqai=hGuQ8kuc9pgc9s8qqaq=dirpe0xb9q8qiLsFr0=vr0=vr0dc8meaabaqaciaacaGaaeqabaqabeGadaaakeaaiiGacuWFYoGygaqcaaaa@2E64@ ~ *N*(*β*, Σ), where ∑=(X˜tX˜)−1σ12+(X˜tX˜)−1σ22
 MathType@MTEF@5@5@+=feaafiart1ev1aaatCvAUfKttLearuWrP9MDH5MBPbIqV92AaeXatLxBI9gBaebbnrfifHhDYfgasaacH8akY=wiFfYdH8Gipec8Eeeu0xXdbba9frFj0=OqFfea0dXdd9vqai=hGuQ8kuc9pgc9s8qqaq=dirpe0xb9q8qiLsFr0=vr0=vr0dc8meaabaqaciaacaGaaeqabaqabeGadaaakeaacqGHris5cqGH9aqpcqGGOaakcuWGybawgaacamaaCaaaleqabaGaemiDaqhaaOGafmiwaGLbaGaacqGGPaqkdaahaaWcbeqaaiabgkHiTiabigdaXaaaiiGakiab=n8aZnaaDaaaleaacqaIXaqmaeaacqaIYaGmaaGccqGHRaWkcqGGOaakcuWGybawgaacamaaCaaaleqabaGaemiDaqhaaOGafmiwaGLbaGaacqGGPaqkdaahaaWcbeqaaiabgkHiTiabigdaXaaakiab=n8aZnaaDaaaleaacqaIYaGmaeaacqaIYaGmaaaaaa@47E6@ is the covariance matrix of the vector *β*.

The Hotelling test is then based on the statistic T2=β^tS−1β^
 MathType@MTEF@5@5@+=feaafiart1ev1aaatCvAUfKttLearuWrP9MDH5MBPbIqV92AaeXatLxBI9gBaebbnrfifHhDYfgasaacH8akY=wiFfYdH8Gipec8Eeeu0xXdbba9frFj0=OqFfea0dXdd9vqai=hGuQ8kuc9pgc9s8qqaq=dirpe0xb9q8qiLsFr0=vr0=vr0dc8meaabaqaciaacaGaaeqabaqabeGadaaakeaacqWGubavdaahaaWcbeqaaiabikdaYaaakiabg2da9GGaciqb=j7aIzaajaWaaWbaaSqabeaacqWG0baDaaGccqWGtbWudaahaaWcbeqaaiabgkHiTiabigdaXaaakiqb=j7aIzaajaaaaa@385B@, with *S *the estimated covariance matrix. Under the null hypothesis of *β *being zero, it follows that

T2p+1~Fp+1,n−2(p+1),
 MathType@MTEF@5@5@+=feaafiart1ev1aaatCvAUfKttLearuWrP9MDH5MBPbIqV92AaeXatLxBI9gBaebbnrfifHhDYfgasaacH8akY=wiFfYdH8Gipec8Eeeu0xXdbba9frFj0=OqFfea0dXdd9vqai=hGuQ8kuc9pgc9s8qqaq=dirpe0xb9q8qiLsFr0=vr0=vr0dc8meaabaqaciaacaGaaeqabaqabeGadaaakeaadaWcaaqaaiabdsfaunaaCaaaleqabaGaeGOmaidaaaGcbaGaemiCaaNaey4kaSIaeGymaedaaiabc6ha+jabdAeagnaaBaaaleaacqWGWbaCcqGHRaWkcqaIXaqmcqGGSaalcqWGUbGBcqGHsislcqaIYaGmcqGGOaakcqWGWbaCcqGHRaWkcqaIXaqmcqGGPaqkaeqaaOGaeiilaWcaaa@424C@

where *p+*1 is the number of parameters in the test, with *p *the degree of the polynomial, and *n *is the number of observations.

### Extension to any number of conditions

The statistical test described in the previous section can be easily extended to the more general case where we wish to compare more than two biological conditions over time. The simplest extension is to perform the same test for any pair of biological conditions. The results of the various pairwise tests can be used to detect, for example, potential biomarkers, by looking at genes with significant *P*-values of one strain compared to all the others. If one is interested in identifying one single list of differentially expressed genes across the conditions, then a simultaneous test is more suited.

To perform a simultaneous test, it is sufficient to test whether the vector of parameters β^=(β^1,…,β^K−1)
 MathType@MTEF@5@5@+=feaafiart1ev1aaatCvAUfKttLearuWrP9MDH5MBPbIqV92AaeXatLxBI9gBaebbnrfifHhDYfgasaacH8akY=wiFfYdH8Gipec8Eeeu0xXdbba9frFj0=OqFfea0dXdd9vqai=hGuQ8kuc9pgc9s8qqaq=dirpe0xb9q8qiLsFr0=vr0=vr0dc8meaabaqaciaacaGaaeqabaqabeGadaaakeaaiiGacuWFYoGygaqcaiabg2da9iabcIcaOiqb=j7aIzaajaWaaSbaaSqaaiabigdaXaqabaGccqGGSaalcqWIMaYscqGGSaalcuWFYoGygaqcamaaBaaaleaacqWGlbWscqGHsislcqaIXaqmaeqaaOGaeiykaKcaaa@3BAE@, with β^j=α^1−α^j+1
 MathType@MTEF@5@5@+=feaafiart1ev1aaatCvAUfKttLearuWrP9MDH5MBPbIqV92AaeXatLxBI9gBaebbnrfifHhDYfgasaacH8akY=wiFfYdH8Gipec8Eeeu0xXdbba9frFj0=OqFfea0dXdd9vqai=hGuQ8kuc9pgc9s8qqaq=dirpe0xb9q8qiLsFr0=vr0=vr0dc8meaabaqaciaacaGaaeqabaqabeGadaaakeaaiiGacuWFYoGygaqcamaaBaaaleaacqWGQbGAaeqaaOGaeyypa0Jaf8xSdeMbaKaadaahaaWcbeqaaiabigdaXaaakiabgkHiTiqb=f7aHzaajaWaaWbaaSqabeaacqWGQbGAcqGHRaWkcqaIXaqmaaaaaa@39C1@, is significantly different from zero. If under the null hypothesis α^1−α^j
 MathType@MTEF@5@5@+=feaafiart1ev1aaatCvAUfKttLearuWrP9MDH5MBPbIqV92AaeXatLxBI9gBaebbnrfifHhDYfgasaacH8akY=wiFfYdH8Gipec8Eeeu0xXdbba9frFj0=OqFfea0dXdd9vqai=hGuQ8kuc9pgc9s8qqaq=dirpe0xb9q8qiLsFr0=vr0=vr0dc8meaabaqaciaacaGaaeqabaqabeGadaaakeaaiiGacuWFXoqygaqcamaaCaaaleqabaGaeGymaedaaOGaeyOeI0Iaf8xSdeMbaKaadaahaaWcbeqaaiabdQgaQbaaaaa@33AA@ and α^1−α^k
 MathType@MTEF@5@5@+=feaafiart1ev1aaatCvAUfKttLearuWrP9MDH5MBPbIqV92AaeXatLxBI9gBaebbnrfifHhDYfgasaacH8akY=wiFfYdH8Gipec8Eeeu0xXdbba9frFj0=OqFfea0dXdd9vqai=hGuQ8kuc9pgc9s8qqaq=dirpe0xb9q8qiLsFr0=vr0=vr0dc8meaabaqaciaacaGaaeqabaqabeGadaaakeaaiiGacuWFXoqygaqcamaaCaaaleqabaGaeGymaedaaOGaeyOeI0Iaf8xSdeMbaKaadaahaaWcbeqaaiabdUgaRbaaaaa@33AC@ are equal to zero, then it follows that α^j−α^k
 MathType@MTEF@5@5@+=feaafiart1ev1aaatCvAUfKttLearuWrP9MDH5MBPbIqV92AaeXatLxBI9gBaebbnrfifHhDYfgasaacH8akY=wiFfYdH8Gipec8Eeeu0xXdbba9frFj0=OqFfea0dXdd9vqai=hGuQ8kuc9pgc9s8qqaq=dirpe0xb9q8qiLsFr0=vr0=vr0dc8meaabaqaciaacaGaaeqabaqabeGadaaakeaaiiGacuWFXoqygaqcamaaCaaaleqabaGaemOAaOgaaOGaeyOeI0Iaf8xSdeMbaKaadaahaaWcbeqaaiabdUgaRbaaaaa@3419@ must be equal to zero too. All one needs to perform the test is to estimate the covariance Σ of the vector β^
 MathType@MTEF@5@5@+=feaafiart1ev1aaatCvAUfKttLearuWrP9MDH5MBPbIqV92AaeXatLxBI9gBaebbnrfifHhDYfgasaacH8akY=wiFfYdH8Gipec8Eeeu0xXdbba9frFj0=OqFfea0dXdd9vqai=hGuQ8kuc9pgc9s8qqaq=dirpe0xb9q8qiLsFr0=vr0=vr0dc8meaabaqaciaacaGaaeqabaqabeGadaaakeaaiiGacuWFYoGygaqcaaaa@2E64@. This can be obtained by considering that var⁡(β^j)=var⁡(α^1)+var⁡(α^j+1)
 MathType@MTEF@5@5@+=feaafiart1ev1aaatCvAUfKttLearuWrP9MDH5MBPbIqV92AaeXatLxBI9gBaebbnrfifHhDYfgasaacH8akY=wiFfYdH8Gipec8Eeeu0xXdbba9frFj0=OqFfea0dXdd9vqai=hGuQ8kuc9pgc9s8qqaq=dirpe0xb9q8qiLsFr0=vr0=vr0dc8meaabaqaciaacaGaaeqabaqabeGadaaakeaacyGG2bGDcqGGHbqycqGGYbGCdaqadaqaaGGaciqb=j7aIzaajaWaaSbaaSqaaiabdQgaQbqabaaakiaawIcacaGLPaaacqGH9aqpcyGG2bGDcqGGHbqycqGGYbGCdaqadaqaaiqb=f7aHzaajaWaaWbaaSqabeaacqaIXaqmaaaakiaawIcacaGLPaaacqGHRaWkcyGG2bGDcqGGHbqycqGGYbGCdaqadaqaaiqb=f7aHzaajaWaaWbaaSqabeaacqWGQbGAcqGHRaWkcqaIXaqmaaaakiaawIcacaGLPaaaaaa@4ADF@ and cov⁡(β^j,β^k)=var⁡(α^1)
 MathType@MTEF@5@5@+=feaafiart1ev1aaatCvAUfKttLearuWrP9MDH5MBPbIqV92AaeXatLxBI9gBaebbnrfifHhDYfgasaacH8akY=wiFfYdH8Gipec8Eeeu0xXdbba9frFj0=OqFfea0dXdd9vqai=hGuQ8kuc9pgc9s8qqaq=dirpe0xb9q8qiLsFr0=vr0=vr0dc8meaabaqaciaacaGaaeqabaqabeGadaaakeaacyGGJbWycqGGVbWBcqGG2bGDdaqadaqaaGGaciqb=j7aIzaajaWaaSbaaSqaaiabdQgaQbqabaGccqGGSaalcuWFYoGygaqcamaaBaaaleaacqWGRbWAaeqaaaGccaGLOaGaayzkaaGaeyypa0JagiODayNaeiyyaeMaeiOCai3aaeWaaeaacuWFXoqygaqcamaaCaaaleqabaGaeGymaedaaaGccaGLOaGaayzkaaaaaa@4357@, again assuming independence between different conditions.

Using the same statistic described in the previous section, it follows that under the null hypothesis of *β *being zero

T2(K−1)(p+1)~F(K−1)(p+1),n−K(p+1),     (4)
 MathType@MTEF@5@5@+=feaafiart1ev1aaatCvAUfKttLearuWrP9MDH5MBPbIqV92AaeXatLxBI9gBaebbnrfifHhDYfgasaacH8akY=wiFfYdH8Gipec8Eeeu0xXdbba9frFj0=OqFfea0dXdd9vqai=hGuQ8kuc9pgc9s8qqaq=dirpe0xb9q8qiLsFr0=vr0=vr0dc8meaabaqaciaacaGaaeqabaqabeGadaaakeaadaWcaaqaaiabdsfaunaaCaaaleqabaGaeGOmaidaaaGcbaWaaeWaaeaacqWGlbWscqGHsislcqaIXaqmaiaawIcacaGLPaaadaqadaqaaiabdchaWjabgUcaRiabigdaXaGaayjkaiaawMcaaaaacqGG+bGFcqWGgbGrdaWgaaWcbaGaeiikaGIaem4saSKaeyOeI0IaeGymaeJaeiykaKIaeiikaGIaemiCaaNaey4kaSIaeGymaeJaeiykaKIaeiilaWIaemOBa4MaeyOeI0Iaem4saSKaeiikaGIaemiCaaNaey4kaSIaeGymaeJaeiykaKcabeaakiabcYcaSiaaxMaacaWLjaWaaeWaaeaacqaI0aanaiaawIcacaGLPaaaaaa@52AA@

where (*K *- 1)(*p *+ 1) is the number of parameters in the test, with *p *the degree of the polynomial and K the number of conditions, and *n *is the total number of observations.

One can derive a similar test to (4) without taking temporal ordering into account. Rather than comparing the coefficients of the quadratic polynomials, the α^c
 MathType@MTEF@5@5@+=feaafiart1ev1aaatCvAUfKttLearuWrP9MDH5MBPbIqV92AaeXatLxBI9gBaebbnrfifHhDYfgasaacH8akY=wiFfYdH8Gipec8Eeeu0xXdbba9frFj0=OqFfea0dXdd9vqai=hGuQ8kuc9pgc9s8qqaq=dirpe0xb9q8qiLsFr0=vr0=vr0dc8meaabaqaciaacaGaaeqabaqabeGadaaakeaaiiGacuWFXoqygaqcamaaCaaaleqabaGaem4yamgaaaaa@2FDE@ in equation (2), the test will now look for differences between the gene expression parameters in the log-ratios equation, the μ^c
 MathType@MTEF@5@5@+=feaafiart1ev1aaatCvAUfKttLearuWrP9MDH5MBPbIqV92AaeXatLxBI9gBaebbnrfifHhDYfgasaacH8akY=wiFfYdH8Gipec8Eeeu0xXdbba9frFj0=OqFfea0dXdd9vqai=hGuQ8kuc9pgc9s8qqaq=dirpe0xb9q8qiLsFr0=vr0=vr0dc8meaabaqaciaacaGaaeqabaqabeGadaaakeaaiiGacuWF8oqBgaqcamaaCaaaleqabaGaem4yamgaaaaa@2FF5@ in equation (1), one for each time point. This means that in equation (4), (*p *+ 1) should now be replaced by the number of time points.

The P-values from all the tests were Benjamini-Hochberg corrected for multiple testing using the multtest package in R.

### Microarray data on muscular dystrophy

Four different mouse strains were included in the study: wild-type controls (C57Bl/10ScSnOlaHsd), dystrophin-deficient mdx (C57Bl/10ScSn ^mdx/J^), beta-sarcoglyan-deficient mice [13] and gamma-sarcoglycan-deficient mice [12]. Animals were sacrificed at the ages of 1, 2.5, 4, 6, 8, 10, 12, 14 and 20 weeks (2 individuals per time point per strain) and RNA was isolated from the quadriceps muscle of these mice. cRNA was prepared by linear amplification and concurrent incorporation of amino-allyl UTP, followed by chemical coupling to monoreactive Cy3 or Cy5 dyes [30]. Labelled targets (1.5 μg cRNA per target) were hybridised to murine oligonucleotide microarrays (65-mer with 5'-hexylaminolinker, Sigma-Genosys mouse 7.5K oligonucleotide library, printed in duplicate).

Prior to the analysis, the microarray data are normalised to account for spatial, dye and across-array effects, using the all.norm function in the smida R package [28,31]. The normalised data are summarised by taking the log-ratios between the two channels on the same array. Microarray data have been submitted to the Gene Expression Omnibus database [32] under series GSE1574 and GSE3523.

### Quantitative PCR experiments

Quantitative RT-PCR was performed as described in [14]. Gene expression levels were calculated using the gene expression macro provided by Bio-Rad (Bio-Rad, Hercules, CA), normalized to glyceraldehyde-3-phosphate dehydrogenase (GAPDH, stable expression in all samples) expression levels, and expressed as log-ratio with respect to time point 1, as was done for the microarrays. Primer sequences are available on request.

## Authors' contributions

V.V. developed the statistical test, wrote the R code and drafted the manuscript. X.L. assisted with the development of the general methodology and of the manuscript. R.T. and E.M. performed the experimental work. P.H. assisted in the biological interpretation of the data and drafted the manuscript. All authors read and approved the final manuscript.

## Figures and Tables

**Table 1:** Biomarker genes for the different muscular dystrophies. Biomarker genes selected at the 5% confidence level from temporal Hotelling pairwise tests, whereby each strain of muscular dystrophy is tested for significant differences with each of the other strains.

Strain	GenBank Accession	*P*-values						Symbol
			
		MDX vs WT	MDX vs BSG	MDX vs GSG	BSG vs WT	GSG vs WT	BSG vs GSG	
MDX	NM_008322	5.12E-06	2.56E-04	3.45E-05	4.52E-01	5.80E-01	7.19E-01	Idh2
	AF192382	1.05E-02	2.20E-03	5.09E-09	6.03E-01	1.30E-03	1.55E-01	Prnd
	NM_016861	3.27E-06	2.56E-04	2.63E-04	6.78E-01	7.60E-01	8.18E-01	Pdliml
	Y13832	3.57E-02	3.02E-02	2.91E-06	2.90E-01	1.79E-05	2.85E-01	Gtl2
	AJ277114	1.63E-03	2.68E-03	2.91E-06	2.30E-01	1.36E-02	5.12E-01	Maged2
	NM_008517	2.58E-04	4.91E-04	3.95E-03	7.06E-01	9.17E-01	3.90E-01	Lta4h
	NM_021467	3.87E-03	2.88E-02	2.49E-02	1.74E-01	8.79E-01	6.31E-01	Tnnil
	NM_011812	3.00E-02	2.66E-02	7.89E-04	9.97E-01	9.98E-01	l.OOE+00	FblnS

BSG	NM_019446	8.24E-01	5.63E-03	7.65E-01	4.15E-02	8.54E-01	2.06E-02	Barhll
	NM_010902	4.65E-01	1.68E-02	6.75E-01	2.19E-03	4.25E-01	6.89E-03	Nfe212
	NM_009536	5.30E-01	2.76E-03	6.46E-01	5.09E-03	9.70E-01	2.06E-02	Ywhae
	NM_020329	7.23E-01	3.02E-02	7.86E-01	3.51E-03	7.98E-01	2.96E-02	Dolppl
	NM_010074	9.57E-01	2.92E-02	4.37E-01	3.04E-02	3.45E-01	3.19E-02	D_pp_4
	NM_016755	9.71E-01	4.26E-02	9.39E-01	4.50E-02	9.83E-01	3.41E-02	Atp5j
	NM_021291	5.10E-01	4.25E-02	6.93E-01	3.25E-02	9.54E-01	4.74E-02	Slc7a9
	J03783	8.29E-01	4.43E-02	9.67E-01	4.53E-02	7.68E-01	2.92E-02	116

GSG	NM.009538	3.72E-01	7.22E-01	2.75E-12	1.67E-01	< 1.00E-12	2.06E-02	Plagll
	NM_015784	9.26E-01	6.22E-02	3.06E-09	l.OOE-02	5.71E-09	3.38E-02	Postn
	NM_016762	7.33E-02	9.23E-01	4.18E-10	7.13E-01	8.45E-04	2.21E-03	Matn2
	M94308	6.28E-01	1.49E-01	1.97E-06	3.32E-01	4.69E-07	4.18E-06	Unknown
	M15442	9.35E-01	8.40E-01	9.02E-07	4.77E-01	2.52E-05	2.90E-03	Plpl
	NM_009079	9.10E-02	8.54E-01	1.30E-05	6.10E-01	4.80E-02	5.89E-03	Rpl22
	AB020886	5.50E-01	8.89E-01	3.10E-04	4.31E-01	1.68E-02	3.38E-02	Akapl2
	NM_007429	5.86E-01	9.90E-01	2.42E-02	7.02E-01	4.75E-04	1.82E-02	Agtr2
	NM_008252	6.77E-01	3.70E-01	8.78E-03	3.31E-01	1.04E-02	2.90E-03	Hmgb2
	AL391037	9.52E-01	9.91E-01	2.92E-02	9.14E-01	2.47E-02	4.23E-02	Palmd
